# Stable C isotope data of southern mixed-grass prairie vegetation from Oklahoma, United States

**DOI:** 10.1016/j.dib.2020.106204

**Published:** 2020-08-21

**Authors:** R.F. Follett, C.E. Stewart, J. Bradford, E.G. Pruessner, Phillip L. Sims, M.F. Vigil

**Affiliations:** a Soil Management and Sugarbeet Research Unit USDA Agricultural Research Service (ARS) Fort Collins CO 80526, U.S.A; b Southern Plains Range Research Station USDA Agricultural Research Service (ARS). Woodward, OK 73801, U.S.A

**Keywords:** Delta 13C, Grazed, Plant diversity, Carbon

## Abstract

Stable carbon isotopic data (δ^13^C) of 41 individual plant species was collected from long-term grazed and ungrazed pastures in Oklahoma, USA. These data can serve as a library of stable carbon isotope values for Southern mixed-grass prairie species. Seventeen warm-season (C_4_) and twenty-four cool-season (C_3_) plants were identified and collected from the United States Department of Agriculture (USDA) Southern Plains Experimental Range (SPER). Plant samples were dried at 55°C, and ground finely. The δ^13^C isotopic compositions were determined using a Europa Scientific automated nitrogen carbon analyzer (ANCA/NT) with a Solid/Liquid Preparation Module (Dumas combustion sample preparation system) coupled to a Europa 20–20 Stable isotope analyzer continuous flow isotope ratio mass spectrometer (Sercon Ltd, formerly Europa Scientific Ltd., Crewe, England). These data can be used as end members in isotopic mixing models or in paleoecology to correlate soil ages with plant species composition. Data from plant species provides information about soil organic carbon sequestration and possible long-term climate change.

## Specifications Table

**Table:** 

Subject	Agricultural and Biological Sciences
Specific subject area	Soil Science
Type of data	Table
How data were acquired	Plant species growing in native and improved pastures under long-term grazed and ungrazed treatments in the United States Department of Agriculture (USDA) Southern Plains Experimental Range (SPER) were identified and collected for analysis to add to a library database of plants found across the US Great Plains. Individual plants were clipped at soil level, dried at 55°C, ground finely. The δ^13^C isotopic compositions were determined using combustion mass spectrometry with each plant sample run in duplicate.
Data format	Raw and analysed data
Parameters for data collection	Individual plant species were collected in long-term grazed and ungrazed pastures near sites of soil sampling. The species represented the diversity of vegetation found in the pastures.
Description of data collection	Several examples of each species were collected as whole plants clipped at the soil surface. Roots were not included. The species were keyed out to confirm the identity. Samples were oven dried at 55°C, and ground finely in preparation for analysis.
Data source location	United States Department of Agriculture (USDA) Southern Plains Experimental Range (SPER), Harper County, Oklahoma United States of America 99° 23’ W, 36° 27’ N
Data accessibility	With the article
Related research article	[2]. Long-term pasture management impacts on eolian sand soils in the southern mixed-grass prairie. Quaternary International. https://doi.org/10.1016/j.quaint.2020.07.019.

## Value of the Data

• Stable carbon isotopes (δ^13^C) are used across a wide variety of disciplines of agricultural, ecological, physiological sciences to infer biological, climate, and soil processes, but are expensive and time-consuming to obtain.
• Researchers in geography, paleoecology, and soil science could use these individual plant species to complement their own work.
• Data from individual plant species provides information about soil organic carbon sequestration and possible long-term climate change.
• These data can be used as end members in isotopic mixing models or in paleoecology to correlate soil ages with plant species composition.

## 1 Data Description

This article contains summarized delta^13^C (δ^13^C) of C_4_ and C_3_ plant species from the Southern Plains Experimental Range, Oklahoma, United States (Table 1). Forty one individual plant species were collected in 2007 and 2008 from long-term grazed and ungrazed pastures comprising seventeen warm-season (C_4_) and twenty-four cool-season (C_3_) plants. Plant samples were run in duplicate and raw data (δ^13^C, percent C percent N) included as supplementary information.

**Table 1:** Delta^13^C (δ^13^C) of C_4_ and C_3_ species from the Southern Plains Experimental Range.

Scientific "latin" name	Common name	δ^13^C PDB
*Cenchrus L.*	Sandbur	-12.04
*Sorghastrum nutans (L.) Nash*	Indiangrass	-12.22
*Andropogon hallii Hack.*	sand bluestem	-12.36
*Panicum virgatum L.*	switchgrass	-12.49
*Bothriochloa ischaemum (L.) Keng*	yellow bluestem	-12.55
*Aristida purpurea Nutt.*	purple threeawn	-12.68
*Eragrostis secundiflora J. Presl*	red lovegrass	-12.72
*Schizachyrium scoparium (Michx.) Nash*	little bluestem	-12.77
*Digitaria cognata (Schult.) Pilg.*	fall witchgrass	-12.78
*Calamovilfa gigantea (Nutt.) Scribn. & Merr.*	giant sandreed	-12.86
*Paspalum setaceum Michx.*	thin paspalum	-12.96
*Eragrostis trichodes (Nutt.) Alph. Wood*	sand lovegrass	-13.09
*Cyperus setigerus Torr. & Hook*	lean flatsedge	-13.14
*Sporobolus cryptandrus (Torr.) A. Gray*	sand dropseed	-13.32
*Bouteloua gracilis (Willd. ex Kunth) Lag. ex Griffiths*	blue grama	-13.49
*Digitaria cognata (Schult.) Pilg.*	common witchgrass	-13.49
*Chloris verticillata Nutt.*	tumble windmill grass	-13.53
*Physalis hispida (Waterf.) Cronquist*	prairie groundcherry	-24.37
*Commelina erecta L.*	whitemouth dayflower	-25.58
*Rhus aromatica Aiton*	fragrant sumac	-25.63
*Prunus angustifolia Marsh.*	Chickasaw plum	-25.77
*Poa arachnifera Torr.*	Texas bluegrass	-26.00
*Conyza canadensis (L.) Cronquist*	Canadian horseweed	-26.21
*Mentzelia nuda (Pursh) Torr. & A. Gray*	bractless blazingstar	-26.37
*Helianthus petiolaris Nutt*	prairie sunflower	-26.53
*Heterotheca villosa (Pursh) Shinners*	hairy false goldenaster	-26.65
*Yucca glauca Nutt. var. glauca*	soapweed yucca	-26.96
*Callirhoe digitata Nutt.*	Cowboy Rose "Wine Cup"	-26.98
*Elymus canadensis L.*	Canada wildrye	-27.03
*Croton glandulosus L*	vente conmigo	-27.05
*Croton texensis (Klotzsch) Müll. Arg.*	Texas croton	-27.37
*Croptilon hookerianum (Torr. & A. Gray) House*	Hooker's scratchdaisy	-27.48
*Artemisia filifolia Torr.*	sand sagebrush	-27.93
*Mirabilis linearis (Pursh) Heimerl*	narrowleaf four o'clock	-28.06
*Ambrosia psilostachya DC.*	western ragweed	-28.26
*Plantago patagonica Jacq.*	woolly plantain	-28.36
*Eriogonum annuum Nutt.*	annual buckwheat	-28.72
*Heterotheca subaxillaris (Lam.) Britton & Rusby*	camphorweed	-29.09
*Ambrosia psilostachya DC.*	Cuman ragweed	-29.09
*Grindelia papposa G.L.Nesom & Suh*	Spanish gold	-29.81
*Gaillardia parryi Greene*	Parry's blanketflower	-29.90

Table 1. The delta^13^C (δ^13^C) of C_4_ and C_3_ plant species from the Southern Plains Experimental Range, Oklahoma, United States.

## 2 Experimental Design, Materials and Methods

Field sampling and design for these data are described in detail in Follett et al. [2]. This study was conducted on the United States Department of Agriculture (USDA) Southern Plains Experimental Range (SPER), 2 km north of Ft. Supply, Harper County, OK (99° 23’ W, 36° 27’ N; elevation 610–640 m). The SPER includes 1,746 ha of native sandhill rangeland (Berg, 1994) that in 1936 was “observed to be in extremely poor condition as a result of severe drought, intense heat, and close grazing.” Current use is for rangeland and pasture research conducted by the United States Department of Agriculture, Agricultural Research Service, Southern Plains Range Research Station, near Woodward, OK. The SPER was established following the historical drought of the 1930’s. In 1941, experimental pastures, ranging from 20 to 80 ha in size and associated exclosures, varying from 0.4 to 4 ha, were fenced and long-term grazing studies began. Between 1940 and 1961, a stocking rate study compared light, moderate, and heavy grazing rates on replicated pastures and determined sustainable grazing levels for this southern plain mixed prairie. Since 1961, the moderately grazed pastures and non-grazed exclosures within grazed pastures have been maintained at these same levels [1]. Improved pastures were planted (1958–1980) to optimize grazing forages with specific plant species. These included both native (switchgrass and sand bluestem) and non-native (weeping lovegrass and caucasian bluestem) species that varied in aboveground productivity.

Individual plant species were collected in long-term grazed and ungrazed pastures and identified to determine plant species composition and isotopic signature. The plants were collected in 2007 and 2008 during the growing season and represent the diversity of species found in the pastures. Forty-one individual plant species were collected, including seventeen warm-season (C_4_) and twenty-four cool-season (C_3_) plants. Plant samples were oven dried at 55°C, and ground finely to pass through a 0.2 mm sieve for C, N and isotope analysis.

The δ^13^C isotopic composition was determined using a Europa Scientific automated nitrogen carbon analyzer (ANCA/NT) with a Solid/Liquid Preparation Module (Dumas combustion sample preparation system) coupled to a Europa 20–20 Stable isotope analyzer continuous flow isotope ratio mass spectrometer (Sercon Ltd, formerly Europa Scientific Ltd., Crewe, England). Data are expressed as δ^13^C relative to the international standard Pee Dee Belemnite.

The δ^13^C was calculated using the equation

$$\delta^{13}C=\left[\frac{13R_{Sample}-13R_{Standard}}{13R_{Standard}}\right]1000$$

where R_sample_ is the ratio of δ^13^C/δ^12^C in the soil and R_standard_ is the ratio of δ^13^C/δ^12^C international Pee Dee Belemnite (PDB).

Samples were run in duplicate and averages of δ^13^C are presented in Table 1.

## Declaration of Competing Interest

The authors declare that they have no known competing financial interests or personal relationships which have, or could be perceived to have, influenced the work reported in this article.

## Appendix Supplementary materials

Image, application 1
